# Editorial: Mechanisms of neurodegeneration in amyotrophic lateral sclerosis and related disorders

**DOI:** 10.3389/fncel.2024.1531449

**Published:** 2024-12-05

**Authors:** Danyllo Oliveira, Agnes Lumi Nishimura

**Affiliations:** ^1^Department of Genetics and Evolutionary Biology, Human Genome and Stem Cell Research Center, Institute of Biosciences, University of São Paulo, São Paulo, Brazil; ^2^School of Medical Sciences Santa Casa and Pathological Sciences Unit, Irmandade Santa Casa de Misericordia de São Paulo, São Paulo, Brazil; ^3^Centre for Neuroscience, Surgery and Trauma, Barts and The London School of Medicine and Dentistry, Blizard Institute, Queen Mary University of London, London, United Kingdom

**Keywords:** amyotrophic lateral sclerosis, neurodegeneration, genetic risk factor, Mendelian randomization, disease mechanisms, spinal muscular atrophy, myopathy

This Research Topic, *Mechanisms of Neurodegeneration in Amyotrophic Lateral Sclerosis and Related Disorders*, includes eight original research papers and two reviews on genetic factors and molecular mechanisms associated with Duchenne muscular atrophy, spinal muscular atrophy, and amyotrophic lateral sclerosis. This Research Topic covers how genetic (*KIF1A* and *TUBBA4A*) and environmental factors (e.g., aging, apnea, osteoporosis, and inflammation) can influence these degenerative conditions. It also explores the disease mechanisms of ALS and drug discovery using zebrafish and patient-derived cells.

We open this editorial with a study investigating the effects of aging on the formation of tubular aggregates. These aggregates are formed upon progressive accumulation of sarcoplasmic reticulum protein, which is often associated with myopathies. de Vasconcelos et al. investigated how degeneration and regeneration in skeletal muscle can influence the presence of these tubular aggregates. The authors investigated wild-type animal models and Duchenne muscular dystrophy models to evaluate the effects of muscle regeneration and tubular aggregate formation. To induce regeneration, the animals were subjected to injury using electroporation and left to recover for 5, 15, and 30 days post-electroporation. The findings revealed that tubular aggregates were more prevalent in aged WT animals than those with aged Duchenne muscular dystrophy. Furthermore, the study showed that the number of tubular aggregates decreased progressively from 5 to 30 days post-electroporation, with the WT-aged animals displaying recovery by 5 days after the injury. The authors propose that tubular aggregates accumulate in muscle fibers as a result of aging and that during the regeneration process, new muscle fibers that do not contain these aggregates are produced. These newly produced muscle fibers do not seem to contribute to an extra functional capacity of the muscle.

Moving on to molecular mechanisms of neurodegenerative diseases, Rashid and Dimitriadi review the pathogenic mechanisms of autophagy and potential therapeutic approaches in spinal muscle atrophy. The authors provide a summary of spinal muscular atrophy, the effect of autophagy on the disease and how alterations in the autophagic flux could be a potential therapeutic route for SMA.

The following studies focused on amyotrophic lateral sclerosis (ALS), a complex condition influenced by genetic and environmental factors. Research has identified various dysregulated proteins in ALS, including those involved in axonal transport. Among these proteins are motor proteins like kinesin, which are essential for transporting cargo, such as vesicles, mRNA, and organelles, from the cell body (soma) to the axon terminals. Mutations in genes associated with axonal transport have been identified in patients with ALS. A recent study discovered mutations in the *KIF1A* gene among ALS patients in southern China, suggesting it may be a genetic risk factor for the disease. To investigate whether *KIF1A* mutations are present in a different cohort of Chinese ALS patients, Zheng W. et al. conducted whole-exome sequencing on 1,068 ALS patients. Of these, 14 patients (1.31%) had mutations in the C-terminal region of the KIF1A gene. Notably, mutations in the motor domain at the N-terminal end of the *KIF1A* gene are linked to hereditary peripheral neuropathy and spastic paraplegia, highlighting that different mutations in this gene can lead to various conditions, thus broadening the spectrum of ALS. This study supports the idea that *KIF1A* mutations are considered a risk factor for ALS within the Chinese population.

The next three studies investigate Mendelian randomization, obstructive sleep apnea, osteoporosis and viral infections. Obstructive sleep apnea (OSA) is a common sleep disorder characterized by reduced hemoglobin oxygen saturation and disrupted sleep due to repeated apneas (pauses in breathing). Previous studies have suggested that patients with amyotrophic lateral sclerosis (ALS) who experience obstructive sleep apnea have worse survival rates compared to those without OSA. OSA can lead to intermittent hypoxia, which negatively affects cells in the central nervous system (CNS), resulting in neuronal injury, increased oxidative stress, and neuroinflammation. This indicates that OSA may contribute to the worsening of ALS symptoms.

Despite the evidence showing that OSA can exacerbate ALS symptoms, it remains unclear whether OSA is a risk factor for ALS. In their study, Du et al. conducted data mining research using Mendelian randomization to explore the relationship between OSA and the risk of developing ALS. The authors analyzed pooled data from genome-wide association studies (GWAS) involving 16,761 OSA patients and 201,194 healthy controls. They also examined meta-analysis data from 22,040 ALS patients and 62,654 healthy controls. By applying the inverse-variance weighted (IVW) method, the authors found that genetic predisposition to OSA was associated with an increased risk of ALS. While the findings should be interpreted with caution, the study highlights how data mining can help identify potential risk factors associated with various conditions.

Next, Li et al. investigated one GWAS dataset with 27,205 ALS cases and 110,881 controls and another GWAS database with 53,236 osteoporosis cases. Bone mineral density in the neck, spine and arm were evaluated. The authors identified 10 qualified SNPs as proxies for ALS. However, no association was observed between osteoporosis and the risk for ALS using IVW. In another study, Zheng Q. et al. conducted a study using Mendelian randomization using the IVW method to investigate a potential association between several viral infections—including herpes simplex virus (HSV), varicella-zoster virus (VZV), Epstein–Barr virus (EBV), cytomegalovirus (CMV), HHV-6, and HHV-7—and ALS. The research analyzed GWAS data from 6,812 subjects affected by these infections and 634,809 controls. The authors concluded that these viruses do not represent a risk factor for ALS.

Deepening on the role of inflammation and innate and adaptive immunity and ALS, Mimic et al. summarize the evidence linking immunity and ALS. In this review, the authors discuss the dual function of astrocytes and microglia as neurotoxic and neuroprotective cells and how other cells play a role in innate and adaptive immunity. The authors also discuss how autoantibodies and cytokines could play a role in the pathogenesis of ALS.

The identification of biomarkers for ALS would have important implications for disease progression, prognosis and treatment effectiveness. Research utilizing ALS biofluids, including serum and cerebrospinal fluid (CSF), has gained popularity in the past decades, although conflicting results have emerged. In this study, Fu et al. investigated the profile of microproteins, albumin, IgG, IgG, index of IgG, albumin quotient, and serum IgG in 870 ALS patients and 96 healthy ALS and healthy controls. The authors observed that approximately one-third of ALS patients in this study showed higher CSF IgG levels. However, CSF IgG index was decreased when compared with healthy controls. Additionally, multiple linear regression analysis indicates that the CSF IgG index is negatively associated with ALS Functional Rating Scale Revised (ALSFRS-R) scores in males with ALS. This suggests that higher levels of CSF IgG are linked to worsening ALS symptoms, leading to an increased risk of mortality in the ALS population. This study suggests that the CSF IgG index could be a potential biomarker for ALS severity.

Closing this Research Topic, two studies delved deeper into ALS disease mechanisms.

Van Schoor et al. evaluated the effects of downregulating the TUBA4A protein in zebrafish. The authors injected an antisense oligonucleotide morpholino into zebrafish and evaluated motor axon morphology and motor behavior (Van Schoor et al.). Downregulation of TUBA4A led to motor axonopathy and motor behavior disturbances, which were rescued by overexpression of wild-type *TUBA4A* mRNA. In addition, the downregulation of TUBA4A altered post-translational modifications of tubulin, acetylation, detyrosination, and polyglutamylation.

Closing this Research Topic, Watts et al. investigated the ER stress response in ALS-patient-derived inducible pluripotent stem cell lines. Three lines were used in this study: one derived from healthy control, one carrying the *TARDBP* G298S mutation, and one carrying the *SOD1* L144F mutation, which differentiated them into motor neurons. These cells were subjected to pharmacologic ER stressors, increasing neuronal death risk. The authors evaluated how thapsigargin and tunicamycin exposure can recapitulate ALS-associated features associated with ER stress, including upregulation of CHOP and BiP and decreased total neurite length. To assess whether pharmacological compounds could rescue ER-stress-related phenotypes, the authors investigate a selective inhibitor of MAP4K4. This kinase can inhibit kenpaullone, a potential compound tested in ALS patients. The MAP4K4 inhibitor increased viability in thapsigargin- and tunicamycin-treated motor neurons. The authors performed phosphoproteomics on MNs treated with ER stressors and MAP4K4 inhibitors and identified JNK, PKC, and BRAF to be differentially expressed in MAP4K4 inhibitor-treated cells. This study highlights the use of patient-derived cells, compound screening, and evaluation of disease-related pathways altered in ALS.

These studies collectively emphasize the importance of investigating how genetic mutations and environmental factors contribute to neurodegenerative diseases. Using animal models and patient-derived cells can offer valuable insights into the mechanisms of these diseases and help identify novel therapeutic approaches for degenerative conditions.

